# Altered sensory system activity and connectivity patterns in adductor spasmodic dysphonia

**DOI:** 10.1038/s41598-020-67295-w

**Published:** 2020-06-23

**Authors:** Tobias Mantel, Christian Dresel, Michael Welte, Tobias Meindl, Angela Jochim, Claus Zimmer, Bernhard Haslinger

**Affiliations:** 1 Department of Neurology, Klinikum rechts der Isar, Technische Universität München, Ismaningerstrasse, 22 Munich, Germany; 2 Department of Neurology, Johannes Gutenberg University, Langenbeckstrasse, 1 Mainz, Germany; 3 Department of Neuroradiology, Klinikum rechts der Isar, Technische Universität München, Ismaningerstrasse, 22 Munich, Germany

**Keywords:** Movement disorders, Dystonia, Sensory processing

## Abstract

Adductor-type spasmodic dysphonia (ADSD) manifests in effortful speech temporarily relievable by botulinum neurotoxin type A (BoNT-A). Previously, abnormal structure, phonation-related and resting-state sensorimotor abnormalities as well as peripheral tactile thresholds in ADSD were described. This study aimed at assessing abnormal central tactile processing patterns, their spatial relation with dysfunctional resting-state connectivity, and their BoNT-A responsiveness. Functional MRI in 14/12 ADSD patients before/under BoNT-A effect and 15 controls was performed (i) during automatized tactile stimulus application to face/hand, and (ii) at rest. Between-group differential stimulation-induced activation and resting-state connectivity (regional homogeneity, connectivity strength within selected sensory(motor) networks), as well as within-patient BoNT-A effects on these differences were investigated. Contralateral-to-stimulation overactivity in ADSD before BoNT-A involved primary and secondary somatosensory representations, along with abnormalities in higher-order parietal, insular, temporal or premotor cortices. Dysphonic impairment in ADSD positively associated with left-hemispheric temporal activity. Connectivity was increased within right premotor (sensorimotor network), left primary auditory cortex (auditory network), and regionally reduced at the temporoparietal junction. Activation/connectivity before/after BoNT-A within-patients did not significantly differ. Abnormal ADSD central somatosensory processing supports its significance as common pathophysiologic focal dystonia trait. Abnormal temporal cortex tactile processing and resting-state connectivity might hint at abnormal cross-modal sensory interactions.

## Introduction

Focal laryngeal dystonia (FLD) is a debilitating task-specific focal dystonia (TSFD) affecting the laryngeal muscles, resulting in phonation impairment that can be classified into a number of overlapping clinical phenotypes^1^. In the most frequent phenotype, adductor-FLD (adductor-type spasmodic dysphonia, ADSD), laryngeal muscle spasms during vocalisation lead to effortful, jerky speech patterns^2^. Previous studies have outlined several functional and structural cortical abnormalities in the disease involving cortices of the phonation network^3^, including altered phonation-related activity using functional MRI (fMRI)^4–6^ or transcranial magnetic stimulation^7^, resting-state connectivity^8^ and grey matter structure^9^ in primary and higher-order (mostly inferior parietal, pre-/supplementary motor) sensorimotor cortices in ADSD. Subcortically, abnormal putaminal structure and connectivity^8,9^ as well as thalamic and cerebellar structure and phonation-related activity^9,10^ have been shown.

Phonation and speech production in the healthy rely on both somatosensory and auditory feedback mechanisms^11,12^. Botulinum neurotoxin type A (BoNT-A) injection into the vocal cords yields temporary symptomatic relief in ADSD by attenuating the symptom-producing muscle spasms (primarily of the thyroarytenoid muscles), yet its possible central role is not well understood^13^. Reduction of phonation-induced electromyographic activity in the bilateral thyroarytenoid muscles in ADSD after unilateral injection with BoNT-A hinted at a possible role of altered sensory feedback from the treated muscle, leading to adapted motor drive also to the non-treated muscle^14^. If one postulates that this represents a central modulation due to peripheral BoNT-A application, it remains to date unresolved if this is guided by modulation of sensory feedback following BoNT-A-induced changes of muscle activity in the injected muscle during phonation, or if there is a direct central BoNT-A effect also present in absence of phonatory activity^13^. BoNT-A-associated alterations of cortical activity have been reported in orofacial non-TSFD during both motor task and somatosensory processing^15,16^, but conflicting observations have been made during phonation in spasmodic dysphonia^4,5^. Further clarification of the relationship of BoNT-A injections and sensory processing in ADSD may inform the development of sensory (e.g. somatosensory, auditory) modulation strategies to complementarily enhance benefit gained from BoNT-A treatment.

Abnormal somatosensory processing and dysfunctional interaction with the motor system is one central aspect in pathophysiologic theories in focal dystonia (FD)^17^. Yet the central patterns of somatosensory stimulus processing abnormalities in the absence of motor task have not been investigated in ADSD, complicated by anatomic constraints in this TSFD. While the principle feasibility of direct laryngeal stimulation was described during magnetencephalography (MEG) in a small group of healthy subjects, several challenges such as its moderate reproducibility, sensitivity to noise/artefacts and considerable efforts regarding both technique and subject training have not yet been sufficiently addressed^18^. Findings in other types of FD suggest that abnormal somatosensory processing may constitute a form of endophenotypic trait^19^. This provides the opportunity to investigate the somatosensory system in ADSD through somatosensory stimulation to more accessible asymptomatic body regions.

This work aimed at characterizing (i) patterns of abnormal central tactile stimulus processing in ADSD, (ii) possible spatial relations between areas of altered stimulation-related activity and dysfunctional (long-range and regional) connectivity at rest, and (iii) possible effects of BoNT-A treatment on the above findings, avoiding confounds by motor execution, BoNT-A-modulated muscle function, or acute compensation mechanisms.

## Methods

### Participants

Fourteen right-handed patients with idiopathic isolated ADSD (PATpre, m/f = 7/7, age 48.0 ± 14.9y; disease duration 6.7 ± 5.6y) were recruited from the hospital movement disorders outpatient clinic in cooperation with a specialized outpatient phoniatric center and compared to a control group of fifteen age- and sex-matched healthy volunteers (CONTR, m/f = 7/8, 46.5 ± 12.3y). Diagnosis of ADSD was established by an experienced phoniatrist, with additional clinical neurological evaluation by a movement disorders-specialised neurologist. Procedures included evaluation of the medical history, phoniatric voice analysis for typical auditive voice/speech abnormalities (e.g. during vocalisations, conversation, standardised text readings), voice acoustic analysis and electroglottography (involving evaluation of jitter, shimmer, and normalized noise energy), microlaryngoscopy and microstroboscopy. We limited our study to ADSD, as it is far more common than the abductor type^2^. Twelve patients (m/f = 7/5, 48.0 ± 13.2y) received regular abobotulinumtoxin injections (total dosage 15.3 ± 5.9 units) in 3–9-month intervals (depending on individual effect duration) with good clinical benefit (as certified by patients‘ subjective reports and the expert impression of the treating phoniatrist), two were BoNT-A-naïve (i.e. have never recieved BoNT-A treatment). Except for the BoNT-A-naïve patients that were only scanned once, patients were scanned twice in pseudo-randomized order: 34.9 ± 7.3d after BoNT-A treatment (PATpost) when their voice was at its best and post-injection breathiness had recovered, and prior to the next BoNT-A treatment (individually, ≥3 months after the last BoNT-A injection) when the BoNT-A effect had clinically waned. The voice handicap index (VHI) was collected at both timepoints to ascertain subjective impairment by dysphonia. The institutional ethics board (Ethikkommission der Technischen Universität München, https://www.ek-med-muenchen.de) approved of the study and all participants gave written informed consent according to the Declaration of Helsinki. All methods were carried out in accordance with relevant guidelines and regulations. All participants had a normal structural MRI scan and, as certified by medical history and a detailed clinical examination by a senior neurologist and movement disorders specialist (C.D.), no additional neuropsychiatric disorders (including no other movement disorders), no sensory deficits, and no neuroleptic drug use; patients had no dystonic symptoms in body regions other than the laryngeal muscles and did not receive voice therapy or medication for dystonia beyond BoNT-A.

### Data acquisition and analysis

MRI acquisition parameters and software for data analysis are detailed in the supplement.

### Tactile stimulation experiment

The tactile stimulation experiment was performed as previously outlined^15,20^. In brief, trains of punctate tactile stimuli (stimulus duration 2 s, jittered inter-stimulus interval of 7–15 s, intensity adapted to local skin sensitivity) were pseudo-randomly applied to forehead (V1, intensity 32 mN), upper lip (V2, 22 mN) and hand (Ha, 45 mN) on either side by von Frey-monofilaments during three fMRI runs using an automated MR-compatible simulation device. A stimulation-induced painless feeling of touch was additionally ensured by mechanical and pain threshold testing in all participants^21^. Preprocessing was performed as in earlier studies employing this stimulation paradigm^15,20^, with an additional inclusion of six head motion parameters as regressors in the first-level general linear model contrasting the individual regressors (stimulation onsets * canonical hemodynamic response function) for the six stimulation conditions (V1, V2, Ha on either side) to the implicit baseline (rest) separately for each condition. One patient was excluded due to suspicion of corrupted stimulation paradigm. Population-based inferences were drawn by introducing the first-level individual contrast images to a second-level flexible factorial model with factors *subject*, *condition* (see above) and *group* (PATpre, PATpost, CONTR) accounting for possible non-sphericity of the error term, with post-hoc planned longitudinal contrasts (PATpre vs. PATpost) applied in case of a significant difference to healthy subjects pre-BoNT-A. A comparison of healthy subjects with patients post-BoNT-A was not primarily performed as no ancillary information was expected compared to the direct within patient group longitudinal analysis. Considering the subtlety of short-time punctate tactile stimuli and the interindividual anatomical variability of sensory representations, significance was determined at a cluster-forming (peak-level) threshold of p < 0.001 with cluster-level multiple comparison-correction at p_FWEc_ < 0.05 (FWE, family-wise error), adjusted for the six conditions (p < 0.0083 (0.05/6)).

### Resting-state experiment

Spatial independent component analysis (sICA) and regional homogeneity (ReHo) analysis were performed on resting-state data to investigate both changes in (i) long-range and (ii) regional functional connectivity (FC). Basic preprocessing of resting-state fMRI (rs-fMRI) scans involved realignment, slice-timing correction, normalization to Montreal Neurological Institute (MNI) space after coregistration with the anatomical scan, followed by linear detrending, regression of six head motion parameters and despiking using AFNI’s (analysis of functional neuroimages) *3dDespike*^22^. One control was excluded from further analysis due to high frame-to-frame motion. The sICA approach reliably separates non-grey matter signal and noise from physiologically meaningful components^23^. For short-range FC analysis, we removed five white matter and cerebrospinal fluid signal principal components in an additional step using the CompCor (component-based noise correction) method, and bandpass filtering (0.01–0.08 Hz) was applied. Image smoothing for regional FC analysis was performed after connectivity measure calculation as recommended^24^.

#### Long-range FC analysis

sICA extracts maximally independent spatial resting-state networks comprising distributed, functionally related (temporally coherent) brain regions by decomposing their linearly mixed signals contained in the spatiotemporal fMRI dataset^25^. Data across participants and conditions were concatenated and stepwise principal component analysis data reduction was performed guided by a prior dimensionality estimation using the minimum description length algorithm, retaining 39 subject-level and 28 group-level components. Data were variance-normalized and z-transformed. The Infomax algorithm was run 100 times in ICASSO^26^ and cluster quality quantified using the index I_q_ mirroring the difference between intra- and extracluster similarity (range 0–1). Direct data back-reconstruction from the aggregate spatiotemporal dataset was performed using GICA I, which is robust for lower model orders^25^. Following the study goal, we then selected three robust (I_q_ > 0.95) lower-model order cortical resting-state networks of interest guided by the spatial pattern of tactile stimulation-induced cerebral activity (figure s-1) for further analysis: the sensorimotor network (SMN), the auditory network (AN) and the task-positive network (central executive network, CEN; figure s-1). Between-group differences were investigated as above using a flexible factorial random effects model (factors *subject*, *group)*. To ensure that only highly connected network regions were analysed, a combined binary mask representing the effect of the within-group analysis (p_FWE_ < 0.05) was applied. Results were peak-level-corrected for multiple comparisons at p_FWE_ < 0.05, adjusted for three investigated networks (p < 0.017 (0.05/3)).

#### Regional FC analysis

ReHo characterizes the local temporal coherence/synchronization of the regional blood oxygen-level dependent signal. The similarity of the time series of each voxel with those of its 26 nearest neighbours (voxels) was calculated in a whole-brain approach, using Kendall’s coefficient of concordance^24^. After z-transformation to increase normality and data smoothing, between-group analysis was performed as above using a flexible factorial random effects model (factors *subject*, *group)*. Following the aim of the study, the statistical analysis was constrained to a combined binary mask of voxels with a robust within-group response to tactile stimulation (p_FWE_ < 0.05) across conditions. Results were peak-level-corrected for multiple comparisons at p_FWE_ < 0.05.

### Regression analyses

Significant associations of severity by VHI and duration of dysphonia with functional activity/connectivity changes were investigated within PAT post-hoc by multiple regression analysis for those analyses showing significant differential between-group activity/connectivity patterns. FWE-multiple comparison corrections were applied as for the respective between-group task/rest analyses and the significance level adjusted for the number of regressors (p < 0.025 (0.05/2)).

## Results

All patients reported a subjective voice improvement after BoNT-A treatment. Accordingly, the VHI improved significantly post BoNT-A (PATpre 72.6 ± 12.4/67.3 ± 19.4 with/without BoNT-A-naïve patients; PATpost 32.3 ± 18.3; Wilcoxon signed-ranks, z = −2.93, p = 0.003). Mechanical detection and pain thresholds and mean perceived intensities did not significantly differ between groups (repeated-measures analyses of variance (rmANOVAs) [*group×condition*], ps > 0.05). Average frame-to-frame motion (rotation and translation) across analysed subject scanning sessions during rest/task fMRI was 0.12 ± 0.046 mm/0.12 ± 0.054 mm and not significantly different between groups (rest: ANOVA, F_2,37_ = 0.94, p = 0.40/task: rmANOVA [*group* × *session*], ps > 0.05).

### Tactile stimulation experiment

Tactile stimulation in patients (pre-/post-BoNT-A) and controls yielded activity in a network involving primary somatosensory cortex (S1), secondary somatosensory cortex (S2), superior parietal cortex, precuneus, intraparietal sulcus, supramarginal gyrus, supplementary motor area, dorsal and ventral premotor cortex, inferior and middle frontal gyrus, insular cortex, superior and middle temporal cortex, anterior cingulate cortices, and thalamus (figure s-1).

**Figure 1:**
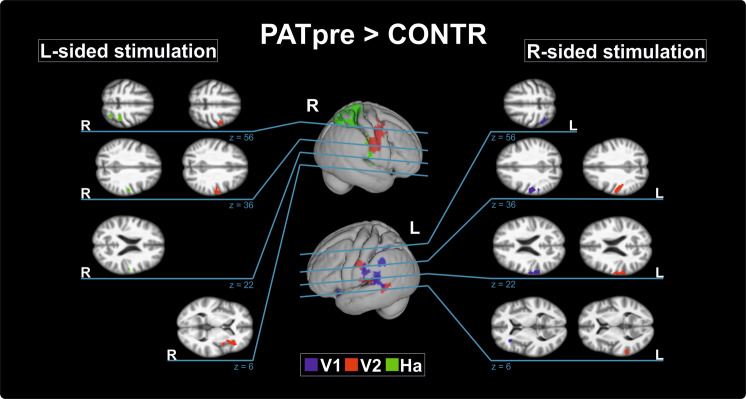
Areas with significantly increased activity in patients with ADSD. The middle column shows differential activation (color coded for each body region) projected on the respective contralateral hemisphere of the participants’ 3D-reconstructed average brain. In the lateral columns, increased activity in in selected areas (top to bottom: S1/superior parietal lobe, S2, insular/temporal cortex) is projected on axial slices of the averaged brain. The overlaid statistical parametric maps were thresholded at p_FWEc_ < 0.0083 and a cluster-forming threshold of p < 0.001 uncorrected. Slice position in MNI space in mm is given relative to the anterior commissure (above +; below −). CONTR, healthy control participants; PATpre, ADSD patients before botulinum toxin A treatment; Ha, dorsal hand; V1, forehead; V2, upper lip, L/R, left/right hemisphere.

Between-group analysis of PATpre against CONTR revealed significantly increased functional activity in the contralateral S1 during left hand/face (V2, Ha) and right face (V1, V2) stimulation, and in the contralateral S2 during right face (V1, V2) stimulation (Table 1, Fig. 1). Further areas of increased contralateral parietal activity in these conditions encompassed the intraparietal sulcus, supramarginal gyrus or superior parietal lobe. Besides enhanced S2 activation, right face stimulation (V1, V2) induced increased temporal activity in the left-hemispheric superior temporal gyrus (STG). Enhanced contralateral insular activity was observed after both left- (V2) and right-sided (V1) stimulus application to the face. Within the frontal lobe, enhanced right ventral (pre)motor (M1/PMv) was seen during left face stimulation (V2), and was visible as trend also in the other conditions in the hemisphere contralateral to stimulation where it did not survive adjustment for the number of conditions (see table s-1 for trends). Post-hoc evaluation of functional activity changes in these conditions before and after BoNT-A within patients did not yield significant differences.

**Table 1:** Areas with stronger activation in patients with ADSD before BoNT-A treatment when compared to healthy controls.

L-sided stimulation						R-sided stimulation					
**V1**						**V1**					
Area	x	y	z	t	V	Area	x	y	z	t	V
−	−					L primary somatosensory, face (BA2)	−54	−20	32	4.78	760
						L supramarginal (BA40)	−60	−34	24	4.58	
						L superior parietal (BA7)	−54	−38	46	4.20	
						L superior temporal (BA22)	−58	−42	16	3.80	
						L secondary somatosensory (OP1)	−64	−20	22	3.63	
						L intraparietal sulcus	−42	−50	58	3.59	
						L superior parietal (BA5)	−32	−52	56	3.21	
						L dorsal insula (BA13)	−32	28	2	4.39	348
						L ventral insula (BA13)	−38	18	−10	4.23	
**V2**						**V2**					
Area	x	y	z	t	V	Area	x	y	z	t	V
R primary somatosensory, face (BA3b)	54	−8	44	4.98	364	L primary somatosensory, face (BA1)	−58	−14	42	4.34	411
R primary motor/ ventral premotor (BA4/6)	42	−4	58	3.79		L primary somatosensory, face (BA2)	−50	−20	36	3.63	
R inferior frontal (BA44)	36	26	0	4.37	346	L intraparietal sulcus (BA40)	−26	−48	42	4.00	
R ventral premotor (BA6)	46	4	10	3.61		L secondary somato- sensory (OP4)	−64	−16	20	4.51	396
R dorsal insula	40	2	6	3.67		L superior temporal (BA22)	−64	−44	14	4.41	
						L supramarginal (BA40)	−64	−32	22	4.28	
**Ha**						**Ha**					
Area	x	y	z	t	V	Area	x	y	z	t	V
R primary somatosensory, hand (BA2)	30	−36	66	4.46	1135	−	−				
R primary somatosensory, face (BA1)	56	−18	44	4.03							
R intraparietal sulcus (BA7)	28	−58	60	3.91							
R superior parietal (BA5/7)	18	−54	64	3.73							
R secondary somatosensory (OP4)	58	−16	18	3.51							
R supramarginal (BA40)	36	−36	42	3.32							

Coordinates (in mm) in the Montreal Neurological Institute space. All differences are significant at p_FWEc _< 0.0083 at a cluster-forming threshold of p < 0.001 uncorrected. BA, Brodmann area; OP, operculum parietale; R, right; L, left; t, t-score; V, cluster volume (voxels).

### Resting state experiment

**Figure 2:**
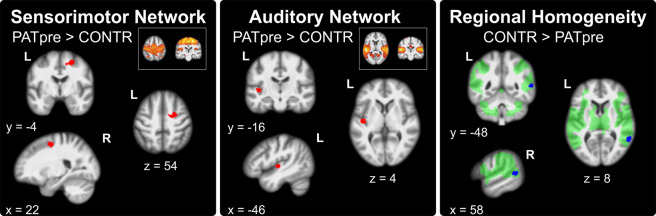
From left to right: Significant increases (p_FWE_ < 0.017) of long-range FC within the sensorimotor and the auditory network (in red) as well as significant reduction of short-range FC by regional homogeneity (p_FWE_ < 0.05; in blue) overlaid onto the participants’ averaged structural images (clusters displayed at p < 0.001 uncorrected); areas with robust within-group response to tactile stimulation across conditions and participants are underlaid in light green. Slice positions in MNI space in mm are given relative to the anterior commissure (right/anterior/above +; left/posterior/below −). CONTR, healthy controls; PATpre, ADSD patients before botulinum toxin A treatment; L/R, left/right hemisphere.

During rest, we observed increased FC in the right medial dorsal premotor cortex (PMd; x | y | z = 24 | −2 | 56; t = 5.26, p = 0.012) within the SMN in PATpre compared to CONTR, increased FC in the left primary auditory cortex (A1; x | y | z = −44 | −14 | 2; t = 5.47, p = 0.004) within the AN in PATpre compared to CONTR and no significant abnormalities in the CEN. We further observed significantly reduced regional homogeneity at the right parieto-temporal junction (TPJ; x | y | z = 60 | −50 | 8; t = 4.97, p = 0.039; Fig. 2) in PATpre compared to CONTR, with a corresponding left-hemispheric trend at k > 50 voxels (x | y | z = −48 | −58 | 14; t = 5.26, k = 86). Post-hoc evaluation of FC changes in these analyses before and after BoNT-A within patients did not yield significant differences.

### Regression analyses

**Figure 3:**
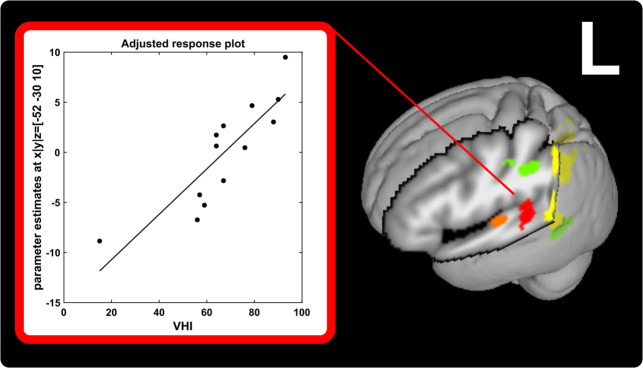
Left-hemispheric temporal cortices showing spatial pattern of abnormal tactile stimulation-induced changes or abnormal resting connectivity. Clusters with a significant positive relation to symptom severity by VHI during right face (V1) stimulation (in red), are displayed together with primary auditory FC-changes within the auditory network (orange) at rest and left-hemispheric temporal (and parietal) activity changes induced by right-hemispheric tactile stimulation of the face (V1/V2, in yellow/green) at a cluster-forming threshold of p < 0.001 uncorrected.

There was a significant positive association of disease severity by voice handicap index with stimulation-induced activation in the left posterior STG (BA41/42; x | y | z = −52 | −30 | 10; t = 6.01, p = 0.009) caudal to A1 during right face (V1) stimulation in PATpre (Fig. 3). All other regression analyses did not yield significant results.

## Discussion

### Abnormal primary somatosensory processing

This work provides evidence for abnormal primary and higher-order somatosensory input processing in ADSD in absence of motor tasks, amending observations in other cranial FDs^15,20,27^. Among studies investigating cranial dystonia, reduced activity during cutaneous somatosensory stimulation was seen in non-task-specific subtypes^15,27^, and increased activity was reported in task-specific forms including the present work^20^. While there is some homogeneity of methodology among those cranial dystonia studies^15,20^, considerable variation of both methodology and directionality of findings is seen in studies in focal dystonias affecting other body parts^28^, and the investigation of cranial cutaneous tactile processing as surrogate to the laryngeal mucosa is a limiting factor regarding the present work. It hence remains uncertain if those variations in directionality might indeed mirror a pathophysiologic difference between dystonia subtypes. In S1, increased activity was observed in the present study during stimulation of both cortically proximate (face) and distant (hand) non-dystonic S1 representations and in both hemispheres, fitting the concept of an underlying endophenotype. Demonstration of such endophenotypic abnormality in cutaneous surrogate areas in ADSD may encourage further research in sensory modulation strategies in the disease. Modulation of sensory input from mostly proximate body regions (i.e. sensory tricks) is known to ameliorate dystonic symptoms in some FD patients, and sensory retraining was able to improve motor function in TSFD of the hand in the past^29^. Quite recent work in FLD already made steps in this direction, indicating positive modulatory effects on voice function through ventral cervical cutaneous vibration^30^, hence the investigation of effects of sensory retraining strategies applied to nondystonic/surrogate sensory areas might be of interest for future work.

Altered cortical activity during left hand stimulation spread lateral into the somatotopic face representations, while increased activity during face stimulation was rather limited to its expected somatotopic representation located superior and posterior to the putative S1 larynx representation reported for the healthy^18^. Increase and spread of somatosensory activation affecting symptomatic and non-symptomatic body parts have been pathophysiologically attributed to deficient intracortical inhibition and dysfunctional plasticity resulting in dedifferentiation and topographic shifts of otherwise concise cortical activation^31–33^. Such cortex-level dedifferentiation in FDs has been suggested to manifest peripherally in altered tactile discrimination thresholds^29^, and altered somatosensory temporal discrimination has also been reported for ADSD^34^. In patients with cervical dystonia, peripheral temporal discrimination thresholds and central correlates of intracortical inhibition were abnormally reduced and further deteriorated by high-frequency somatosensory stimulation of a non-dystonic body part, whereas the opposite effect was seen in healthy controls^35^. As observed for a body part not affected by dystonic posturing, this observation had been discussed in support of a primary rather than adaptive nature of abnormal somatosensory processing and underlines the vulnerability of such predisposing abnormality to intensive somatosensory input as it occurs during high-proficiency motor tasks, as are affected in TSFDs like ADSD.

### Abnormal higher-order sensory processing

Beyond S1, higher-order somatosensory cortices encompassing the left-hemispheric S2 as well as left- and right-hemispheric superior and inferior parietal cortex showed increased tactile-stimulation induced activity in ADSD. Further, right-hemispheric PMv activity was enhanced during left face stimulation (with respective contrahemispheric trends observed during stimulation of other body parts), and medial PMd connectivity was increased at rest. Other rs-fMRI studies in spasmodic dysphonia described reduced connectivity in either the supplementary motor area or the PMd within the SMN. Yet, cluster locations differed compared to the present study, and the lack of dimensionality information in previous studies impairs comparability^8,36^. Disordered information transfer from the sensory to the motor cortices (i.e sensorimotor integration) is discussed as a possible mechanism of dystonic posturing^17^, occurring either at the cortico-cortical or subcortico-cortical level involving the basal ganglia or cerebellum^37,38^. In support of a possible top-down process (from those cortices to primary/subcortical areas), recent work in spasmodic dysphonia suggested abnormal premotor-parietal-putaminal circuitry with abnormal excitatory left inferior parietal projections to the putamen and interhemispheric information transfer from the right to left premotor cortex^36^. Yet, the lack of differentiability of primary and adaptive changes by fMRI ultimately limited the interpretation of the findings’ origins. Past studies in other FDs have attempted to modulate sensorimotor higher-order axis processing through transcranial stimulation paradigms (mostly of the premotor cortex), showing mixed and mostly short-term results on motor function; yet such studies are to date lacking in FLD^39^.

### Temporoparietal interface

An interesting aspect of this study was the observation of temporal cortex and temporoparietal junction abnormalities in absence of phonation. Cortical activity after right-sided face stimulation was increased in the left posterior STG. In parts of the left posterior STG, the impairment (by VHI) further predicted the degree of right face stimulation-induced activity in patients, amending a prior structural analysis that observed a positive relationship of left superior temporal cortical thickness with the mean number of voice breaks in sporadic spasmodic dysphonia^40^. Given that patients in the present study did not undergo a complementary external rating of their disease-related voice impairment (e.g. based on a Likert scale or scorings such as the Unified Spasmodic Dysphonia Rating Scale), this association warrants confirmation in future studies. Additionally, FC of the left A1 cortex within the auditory network was increased at rest, and regional FC in the parieto-temporal junction at rest was disturbed in the right hemisphere with a trend for the left hemisphere (possibly owed to right-hemispheric dominance of processing at the TPJ^11^). Associations of disease duration with functional connectivity, hinted at by previous work in FLD reporting inverse correlation of disease duration with SMN parietal connectivity^41^, were not observed in this work. The above primary/associative (superior) temporal regions have been suggested to be part of primary areas within the phonation network besides the larynx motor cortex, associated PMv, supplementary motor area and cerebellum^3^, that guides speech processing modulated by somatosensory/auditory feedback loops^12^ which have been shown to interact e.g. during stabilisation of the fundamental frequency^42^. Cross-modal interplays within the sensory system have recently gained increasing attention in the healthy. Multisensory processing may occur at different hierarchical levels of the respective sensory system and/or may modulate the sensory processing in classical (primary) stimulus-specific areas via feedback mechanisms^43,44^. Especially a role of regions in and around (posterior to) the auditory cortex has been suggested of relevance in this regard using event-related potentials in humans and intracranial recording in macaques^43,44^. With regard to audiotactile processing, fMRI studies have shown considerable spatial overlap of tactile and auditory stimulation-induced cortical networks with possible frequency-specific components^45,46^. A MEG study described modulation of somatosensory processing speed in the healthy in the presence of auditory stimuli^47^. Cross-modal convergence may occur at the cortico-cortical or subcortical (e.g. superior colliculi) level^43^. Disturbed neuronal synchrony by regional homogeneity in the TPJ may hint at disturbed intracortical processing in this multimodal area. Impaired sensory processing beyond the somatosensory system is yet little researched in FD. Nevertheless, impaired visuo-cerebellar (seed-based/ between-network) connectivity profiles have been described in cranial FDs, and for musician’s embouchure dystonia auditory network abnormalities at rest have previously been shown^48^, hinting at a possible subtype-associated component. Elucidating the effects of possible cross-modal dysfunctional interactions may hence be of interest in future studies aiming at further elucidating FD pathophysiology or between-subtype differences. Further, in analogy to somatosensory retraining approaches, this might incent the development of auditory (re)training approaches to ameliorate FLD symptoms, e.g. based on auditory perceptual observations such as an abnormal Lombard effect previously observed in ADSD patients^49^.

### Absence of overlapping differential activity and connectivity profiles

While both connectivity and stimulation-induced activation abnormalities affected the premotor cortex and upper parts of the temporal cortex, we did not observe spatial overlap. Premotor cortices were abnormally active in the ventral domain during tactile processing, while task-free connectivity was altered dorsally. In the upper temporal cortex, changes differed in their anterior-posterior location (Fig. 3). While some resting state networks seem to display a degree of task/event-related co-activation^50^, the degree to which (parts of) certain intrinsic connectivity networks are recruited for^50,51^ and during^52^ a task or intervention may be varying and undergo dynamic change. Hence, beyond methodological aspects (e.g. sample size, network dimensionality) these observations may point to potential context-related dynamics in the disease whose relevance needs to be better understood to gain further insights into the complex focal dystonia pathophysiology, and may warrant cautious study design in interventional studies aiming at focal non-invasive (transcranial) modulation of cortical activity to ameliorate motor function as discussed above.

### Absence of cortical BoNT-A-related change

In the present study we did not find evidence for BoNT-A-induced modulation of abnormal cortical processing. Methodological aspects such as small sample size, small applied average doses (Ø 15 units Abobotulinumtoxin) and the fact that the larynx representation was not directly tested may have resulted in reduced sensitivity to detect those changes. Alternatively, the probably endophenotypic changes in non-symptomatic representations might mirror underlying predisposing pathophysiology and hence not be responsive to BoNT-A, indicating that modulation of sensory input through BoNT-A observed in earlier clinical work^14^ might indeed rather be closely linked to its modulation of muscle activity. Prior investigations of BoNT-A effects on phonation-induced brain activity were equivocal in spasmodic dysphonia^4,5^. Other studies investigating BoNT-A effects focused on craniocervical non-TSFDs and reported varying sensorimotor system effects^13^.

## Conclusion

The present study provides evidence for abnormally organized somatosensory processing in ADSD amending previous observations of abnormal peripheral tactile discrimination thresholds in the disease. Beyond supporting the notion of dysfunctional sensory processing as common pathophysiologic trait across FD subtypes, evidence of abnormal central somatosensory processing in cutaneous surrogate areas may serve as working point regarding sensory modulation strategies in the disease. Temporal cortex abnormalities during rest and tactile stimulus processing might hint at abnormal cross-modal sensory interactions warranting further research.

## Supplemenatry information

Supplemenatry information.
