# Supplementary material for: Altered sensory system activity and connectivity patterns in adductor spasmodic dysphonia
Source: Sci Rep. 2020 Jun 23;10:10179. doi: 10.1038/s41598-020-67295-w (PMC7311401; doi:10.1038/s41598-020-67295-w)
Supplement: Supplementary file 1 — Supplemenatry information. [file 41598_2020_67295_MOESM1_ESM.pdf]

Supplementary material

Altered sensory system activity and connectivity patterns in adductor spasmodic dysphonia

Tobias Mantel, MD[1]; Christian Dresel, MD[1,2]; Michael Welte, MD[1]; Tobias Meindl, MD[1]; Angela Jochim, MD[1]; Claus Zimmer, MD[3] and Bernhard Haslinger, MD[1]*

[1]Department of Neurology, Klinikum rechts der Isar, Technische Universität München, Ismaningerstrasse 22, Munich, Germany

[2]Department of Neurology, Johannes Gutenberg University, Langenbeckstrasse 1, Mainz, Germany

[3]Department of Neuroradiology, Klinikum rechts der Isar, Technische Universität München, Ismaningerstrasse 22, Munich, Germany

***Correspondence to:** Prof. Dr. Bernhard Haslinger, Klinik und Poliklinik für Neurologie, Klinikum rechts der Isar, Technische Universität München, Ismaninger Strasse 22, D - 81675 München, Germany; E-Mail (corresponding author): bernhard.haslinger@tum.de; Tel 0049-89-4140-4672)

Supplementary methods: data acquisition and software

Data were acquired on a 3T Achieva scanner (Philips N.V., the Netherlands) using an 8-channel head coil. Participants were instructed to keep their eyes closed during the whole experiment, concentrate on the tactile stimuli during the task runs (encouraged by announcing a post-fMRI questionnaire[1,2]) and the head was fixed with foam pads to minimize the risk of motion artefacts. One resting-state run, and subsequently three runs of experimental tactile stimulation at each 303 T2*-weighted echo planar scans (repetition time/echo time (TR/TE) 2200/30ms, field of view (FoV) 216x216 mm^2, 36 slices, slice gap 0.5mm, voxel size 3x3x3mm^3) were collected. Additionally, a high-resolution 3D T1-weighted scan was acquired as anatomical reference (TR/TE/TI=9/4/780ms, FoV=240x240mm^2, voxel size 1x1x1mm^3, 170 slices).

Functional data were analysed using SPM12 (https://www.fil.ion.ucl.ac.uk/spm), DPABI routines (v3.0, http://rfmri.org/DPARSF) and GIFT (v3.0a; http://mialab.mrn.org/software/gift) for Matlab2016a (MathWorks Inc., USA). Deformation parameters for normalization of functional data were calculated from structural data following coregistration using CAT12 (http://dbm.neuro.uni-jena.de/cat/).

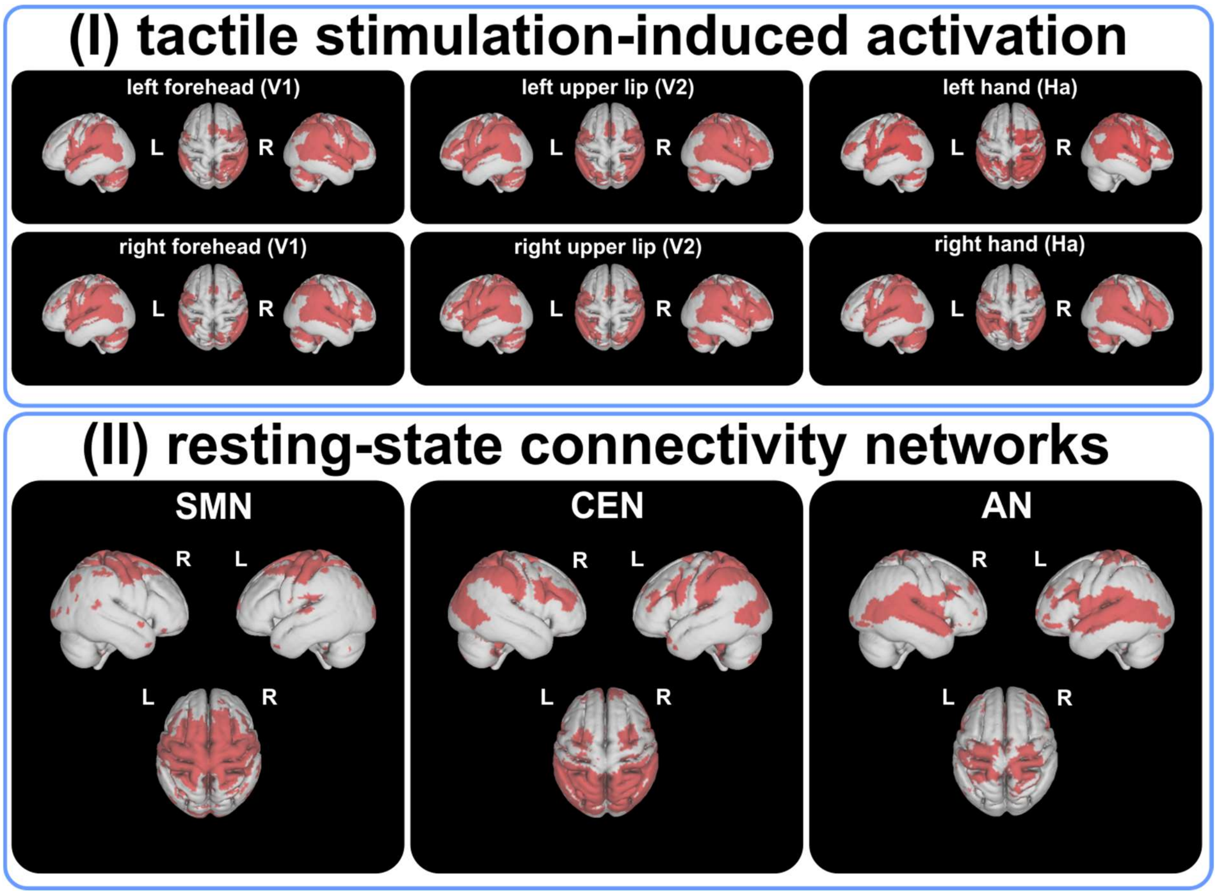

Figure s-1. Tactile stimulation-induced cortical activity networks (I) and resting-state connectivity networks (II) across all study participants, tresholded at p<.001 uncorrected, projected on the 3D reconstructed anatomical scan of all participants in MNI space. SMN, sensorimotor network; CEN, central executive network; AN, auditory network; L/R, left/right hemisphere.

Table s-1. Areas with trends (k>50 voxels) towards stronger activation in patients with ADSD before BoNT treatment when compared to healthy controls

**L-sided stimulation**						**R-sided stimulation**					
**V1**						**V1**					
**Area**	**x**	**y**	**z**	**t**	**V**	**Area**	**x**	**y**	**z**	**T**	**V**
–			–			**R primary motor, face (BA4)**	**-40**	**-8**	**56**	**4.24**	**267**
						**R dorsal premotor (BA6)**	**-32**	**-4**	**50**	**3.68**	
						**R dorsal insula (BA13)**	**36**	**12**	**0**	**4.08**	**223**
						**R dorsal insula**	**38**	**28**	**-2**	**3.68**	
						**L inferior frontal (BA44/45)**	**-50**	**12**	**24**	**4.07**	**319**
						R caudate nucleus	16	6	6	5.01	92
						L inferior parietal sulcus (BA5)	-32	-40	40	4.71	176
						L caudate nucleus	-14	2	14	3.97	107
						L thalamus	-10	-12	18	3.85	
						L inferior frontal sulcus (A45/46)	-50	38	18	3.91	50
						R middle frontal (BA10)	38	-8	48	3.81	75
						L primary motor (BA4)	-26	-22	66	3.78	56
						R inferior parietal (BA40)	64	-40	22	3.72	55
						R inferior frontal (BA44)	44	14	28	3.40	51
						R ventral premotor (BA6)	42	8	36	3.31	
**V2**						**V2**					
**Area**	**x**	**y**	**z**	**t**	**V**	**Area**	**x**	**y**	**z**	**T**	**V**
**R ventral premotor/ inferior frontal (BA6/44)**	**60**	**8**	**18**	**4.10**	**215**	L primary motor, face (BA4)	-40	-8	54	4.13	168
R ventral premotor (BA6)	46	8	44	4.22	78	L ventral premotor (BA6)	-30	-2	54	3.55	
L secondary somatosensory (OP4)	-62	-18	20	4.03	68	R primary somatosensory, face (BA1/2)	56	-16	40	3.77	81
L primary somatosensory, face (BA1)	-58	-20	38	3.96	130	L ventral premotor/ inferior frontal (BA6/44)	-50	12	24	3.69	54
L superior temporal (BA22)	-52	0	0	3.61	56	L primary somatosensory (BA1/2)	-26	-38	66	3.64	70
L inferior frontal (BA44)	-50	2	10	3.40		L superior parietal (BA5)	-22	-44	74	3.19	
						L inferior parietal (BA39)	-48	-74	20	3.51	81
						L parietotemporal junction (BA37/39)	-50	-64	12	3.23	
**Ha**						**Ha**					
**Area**	**x**	**y**	**z**	**t**	**V**	**Area**	**x**	**y**	**z**	**T**	**V**
**R ventral premotor (BA6/44)**	**48**	**8**	**28**	**4.16**	**288**	**L ventral premotor (BA6/44)**	**-54**	**12**	**26**	**3.99**	**290**
**R inferior frontal junction (BA6/8/44)**	**44**	**8**	**42**	**3.20**		**L inferior frontal junction (BA6/8/44)**	**-42**	**4**	**48**	**3.45**	
R dorsal premotor (BA6)	32	-2	58	4.18	138	**R primary motor (BA4)**	**-40**	**-8**	**54**	**3.25**	
L secondary somatosensory (OP4)	-60	-18	22	4.08	84	L intraparietal sulcus (BA7)	-30	-54	42	3.99	115
L superior parietal (BA5)	-30	-38	48	3.95	147	L ventral insula (BA13)	-30	22	-6	3.87	65
L primary motor (BA4/6)	-56	8	32	3.66	200	L primary somatosensory, hand (BA2)	-26	-38	54	3.80	64
L ventral premotor (BA6/44)	-44	8	34	3.65		L superior temporal (BA22)	-60	-42	12	3.79	87

Trends are depicted k>50 voxels at a cluster-forming threshold of p<.001 uncorrected. Clusters that were significant at p_{FWEc}<.05 but did not survive correction for the number of conditions are highlighted in bold.
Coordinates (in mm) in the Montreal Neurological Institute space. BA, Brodmann area; OP, operculum parietale; R, right; L, left; t, t-score; V, cluster volume (voxels).

References (supplement)

1 Dresel, C., Haslinger, B., Castrop, F., Wohlschlaeger, A. M. & Ceballos-Baumann, A. O. Silent event-related fMRI reveals deficient motor and enhanced somatosensory activation in orofacial dystonia. *Brain : a journal of neurology* **129**, 36-46, doi:10.1093/brain/awh665 (2006).

2 Mantel, T. *et al.* Activity and topographic changes in the somatosensory system in embouchure dystonia. *Movement disorders : official journal of the Movement Disorder Society* **31**, 1640-1648, doi:10.1002/mds.26664 (2016).
